# Highly Efficient and Selective Extraction of Gold from Thiosulfate Leaching Solution Using Functionalized Dicationic Ionic Liquids

**DOI:** 10.3390/molecules29112659

**Published:** 2024-06-04

**Authors:** Qiang Zhou, Yunchang Fan, Sheli Zhang

**Affiliations:** 1 College of Chemistry and Chemical Engineering, Henan Polytechnic University, Jiaozuo 454003, China; 212112010027@home.hpu.edu.cn; 2 School of Science and Technology, Jiaozuo Teachers College, Jiaozuo 454000, China; 1295006035@jzsz.edu.cn

**Keywords:** functionalized dicationic ionic liquids (DILs), extraction, thiosulfate leaching, gold recovery

## Abstract

Thiosulfate leaching has been regarded as a promising alternative to cyanidation, but it still faces the challenge of the recovery of low content of gold from high concentrations of thiosulfate solutions. Liquid–liquid extraction is a method to address this issue but is still limited by the use of volatile and toxic organic solvents. To overcome this limitation, this work synthesized some functionalized dicationic ionic liquids (DILs) to serve as extraction solvents for the recovery of the gold–thiosulfate complex, [Au(S_2_O_3_)_2_]^3−^, from thiosulfate solutions. Experimental results indicated that the DILs showed higher extraction rates toward [Au(S_2_O_3_)_2_]^3−^ compared with their monocationic-based counterparts, likely due to the stronger electrostatic interaction between the dications of the ILs and [Au(S_2_O_3_)_2_]^3−^. The transfer of [Au(S_2_O_3_)_2_]^3−^ from the water phase to the IL phase was identified as an anion exchange and endothermic process. The rate of extraction was limited by the anion exchange process occurring at the IL–water interface. The extraction ability of ILs highly depended on the type of anion; specifically, the ILs with anions that had strong hydrogen-bonding ability exhibited high extraction ability toward [Au(S_2_O_3_)_2_]^3−^. Finally, DILs proved effective in the recovery of [Au(S_2_O_3_)_2_]^3−^ from an actual gold leaching solution and exhibited high selectivity toward coexisting ions, indicating their potential as environmentally friendly solvents for gold recovery.

## 1. Introduction

Cyanidation is the most widely used hydrometallurgical technique worldwide for the recovery of gold from gold ores due to its cost-effectiveness and efficiency [1,2]. However, the high toxicity of cyanides has led to a pressing need for more environmentally friendly alternatives. Thiosulfates have emerged as a promising substitute for cyanides in gold leaching due to their non-toxic and effective properties [1,2,3,4,5]. Despite the environmental benefits of the thiosulfate leaching method, the recovery of a low level of gold from the leaching solution containing a high concentration of thiosulfate [1,2,4] remains a significant challenge. Various methods such as adsorption with anion exchange resin [6], activated carbon [7], and silica gel [8], as well as solvent extraction [9,10,11], have been employed to recover gold from thiosulfate solutions. Solvent extraction is considered a preferable option for gold recovery due to its continuous operation, high capacity, and ease of scaling up for industrial use [9,10,11]. Very recently, Li and coworkers reported the extraction of gold–thiosulfate complex, [Au(S_2_O_3_)_2_]^3−^, from a thiosulfate solution with diphenylphosphine (DPP) diluted in benzene, and it was found that DPP demonstrated high selectivity and extraction ability toward [Au(S_2_O_3_)_2_]^3−^, and the extraction mechanism involved the complexation between phosphorus atom in DPP and gold ion via ligand exchange [9]. Furthermore, no research has been carried out to verify the purity of gold after extraction. Despite the high efficiency of the DPP–benzene system in recovering gold, the use of benzene as a solvent is a concern due to its volatility and toxicity. Therefore, there is an urgent demand to develop more environmentally friendly solvents.

Ionic liquids (ILs), a novel class of versatile solvents, have been introduced as a promising alternative to traditional solvents for the extraction of valuable metals like gold [10,11] silver [12], palladium [13], rhodium [14], copper [12], and platinum [14]. Ammonium, imidazolium, and phosphonium-based ILs have been commonly used in the past two decades due to their high chemical stability, non-flammability, and low toxicity [10,11,12,13,14]. Dicationic ILs (DILs) are a new class of the IL family and composed of one dication formed by two monocations connected via a linkage chain, and two anions [15]. DILs have been used for the extraction of Pb^2+^ and Cd^2+^ and have demonstrated higher metal extraction ability compared with the monocationic ILs, as they have more active interaction sites with metals [15,16]. The extraction mechanisms of metals using ILs as extractants typically rely on the IL solubility in water, with hydrophobic ILs following an ion exchange mechanism and more hydrophilic ILs, leading to an ion-pair mechanism [10,11,12,13,14,15,16,17]. Several reviews have highlighted the various applications of ILs in metal extraction [17,18,19].

Up to now, the extraction of gold from thiosulfate leaching solutions by ILs has been reported by Mahandra and coworkers. They constructed an extraction system using Cyphos IL 101 (tetradecyl-(trihexyl) phosphonium chloride) or Cyphos IL 102 (tetradecyl-(trihexyl) phosphonium bromide) as extractant and toluene as a diluent to recover gold from thiosulfate leaching solutions [10,11]: The gold–thiosulfate complex [Au(S_2_O_3_)_2_]^3−^ moves into the organic phase via an anion exchange process, and the ionic interaction between the IL cation and [Au(S_2_O_3_)_2_]^3−^ plays an important role during extraction. Finally, the gold extracted into the organic phase could easily be stripped using a NaCl solution or a solution containing 5% thiourea and 1% HCl. While promising outcomes have been achieved using ILs for gold recovery, the use of volatile and toxic toluene as a solvent remains a necessity. Hence, there is a pressing need to develop an IL-based extraction system that eliminates the reliance on such hazardous solvents. Since the ionic interaction between the IL cation and the anion [Au(S_2_O_3_)_2_]^3−^ facilitates the transfer of gold from the water phase into the organic phase, it is suggested that DILs with highly electrophilic cations could exhibit a strong affinity toward [Au(S_2_O_3_)_2_]^3−^.

Inspired by the above discussion, a series of imidazolium-based DILs were designed, and the aims of this work were thus to investigate their extraction ability toward [Au(S_2_O_3_)_2_]^3−^, explore the extraction mechanism, and assess the potential use of DILs for the recovery of gold from thiosulfate leaching solutions in gold ore processing.

## 2. Results and Discussion

### 2.1. Screening of the ILs

**Figure 1:**
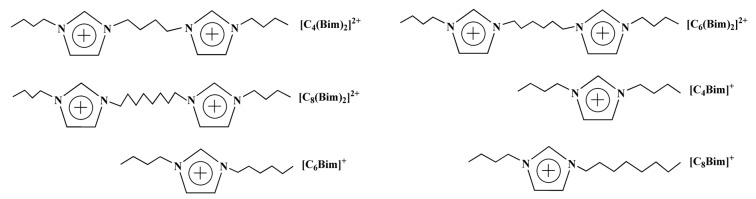
Chemical structures of the IL cations used in this work.

In this work, twelve monocationic and dicationic ILs were synthesized (Figure 1). Among them, [C_4_(Bim)_2_](CF_3_SO_3_)_2_, [C_6_(Bim)_2_](ClO_4_)_2_, [C_6_(Bim)_2_](CF_3_SO_3_)_2_, and [C_8_(Bim)_2_](ClO_4_)_2_ are solid at room temperature with melting points ranging from 54.8 °C to 57.7 °C, 46.4 °C to 48.1 °C, 55.8 °C to 56.4 °C, and 85.7 °C to 87.1 °C, respectively. Operating extraction processes above the melting points of these four solid DILs is not recommended due to the high energy consumption associated with heating. Additionally, the remaining eight ILs also contain ClO_4_^−^- and CF_3_SO_3_^−^-based DILs, and the four solid DILs are not utilized in subsequent extraction procedures.

**Figure 2:**
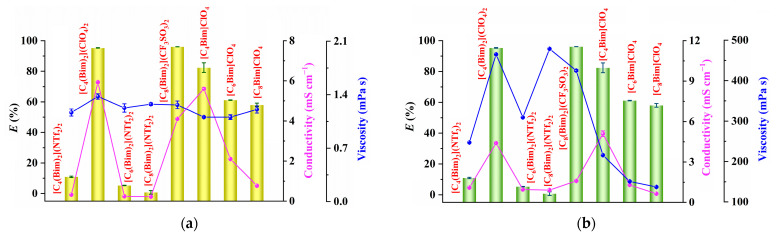
(**a**) Correlation between the extraction ability of ILs and the conductivities of water phases saturated with ILs (purple line) or the viscosities of water phases saturated with ILs (blue line); (**b**) correlation between the extraction ability of ILs and the conductivities of ILs saturated with water (purple line) or the viscosities of ILs saturated with water (blue line). Extraction conditions: 10 mg dm^−3^ of gold, 0.1 mol dm^−3^ of Na_2_S_2_O_3_, 1.0 mol dm^−3^ of NH_3_, 5 mmol dm^−3^ of CuSO_4_, 5:1 of volume ratio of water phase to IL phase (*V*_W_:*V*_IL_), 25 °C of extraction temperature, and 20 min of extraction time.

The extraction ability of the eight different ILs toward [Au(S_2_O_3_)_2_]^3−^ was systematically investigated, and the results illustrated in Figure 2 suggest that the ILs with NTf_2_^−^ anions have low extraction efficiencies for [Au(S_2_O_3_)_2_]^3−^, while those with ClO_4_^−^ and CF_3_SO_3_^−^ anions demonstrate high extraction efficiencies. Typically, the extraction of metal ions with hydrophobic ILs involves three main steps: migration of metal ions from the bulk aqueous phase to the water–IL interface, charge neutralization at the IL–water interface through ion exchange or ion-pair mechanisms, and the diffusion of metal ions into the IL phase [20]. Factors such as conductivity and viscosity play a role in the diffusion rates of metal ions, with high conductivity and low viscosity being favorable for fast diffusion [21,22]. The results depicted in Figure 2a indicate that water phases saturated with ILs have low viscosities similar to the viscosity of water itself (approximately 1 mPa s [23]), yet ILs with ClO_4_^−^ and CF_3_SO_3_^−^ anions still exhibit high extraction efficiencies for [Au(S_2_O_3_)_2_]^3−^ despite their high viscosities (Figure 2b). This suggests that viscosity may not be the primary factor influencing the extraction of [Au(S_2_O_3_)_2_]^3−^. Moreover, water phases saturated with [C_4_(Bim)_2_](ClO_4_)_2_ and [C_4_Bim]ClO_4_, as well as [C_4_(Bim)_2_](ClO_4_)_2_ and [C_4_Bim]ClO_4_ phases saturated with water, show higher conductivities. These two ILs also show stronger extraction efficiencies toward [Au(S_2_O_3_)_2_]^3−^, indicating the rapid diffusion of [Au(S_2_O_3_)_2_]^3−^ in highly conductive media. Additionally, the conductivity of [C_8_Bim]ClO_4_ is similar to the NTf_2_^−^-based ILs, but its extraction ability for [Au(S_2_O_3_)_2_]^3−^ is significantly higher (Figure 2b). This suggests that conductivity is not the sole determining factor in the extraction of [Au(S_2_O_3_)_2_]^3−^.

**Figure 3:**
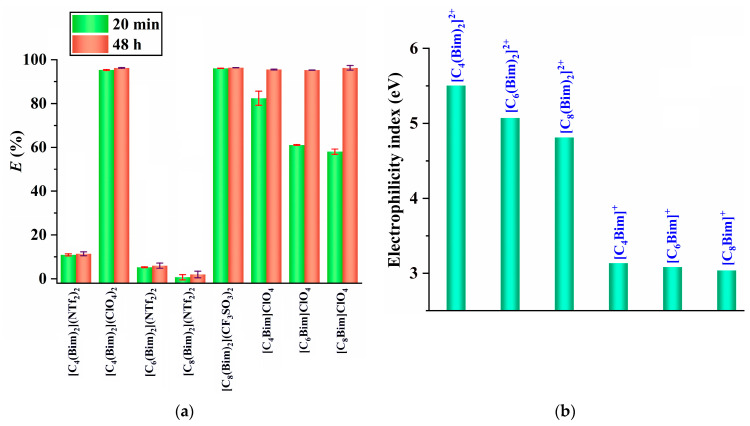
(**a**) The extraction efficiencies of ILs at different extraction times; (**b**) the electrophilicity of ILs.

Based on the above discussion, it appears that the charge neutralization of [Au(S_2_O_3_)_2_]^3−^ at the water–IL interface is the step that determines the rate of the extraction process. To delve deeper into the extraction kinetics, further experiments were carried out by extending the extraction time to 48 h. The results presented in Figure 3a demonstrate that the extraction efficiencies of the monocationic ILs including [C_4_Bim]ClO_4_, [C_6_Bim]ClO_4_, and [C_8_Bim]ClO_4_ increase remarkably with longer extraction times, from 20 min to 48 h. However, the extraction efficiencies of the DILs remain constant when the extraction time is extended from 20 min to 48 h. This suggests that the charge neutralization of [Au(S_2_O_3_)_2_]^3−^ at the water–DIL interface occurs more rapidly compared with the water–monocationic IL interface, likely due to the higher electrophilicity of the dications, as depicted in Figure 3b. In other words, dicationic structure allows for more active interaction with the [Au(S_2_O_3_)_2_]^3−^ anion, leading to quicker charge neutralization through ion exchange. This ion exchange mechanism will be further elaborated in subsequent discussion (Section 2.3).

**Figure 4:**
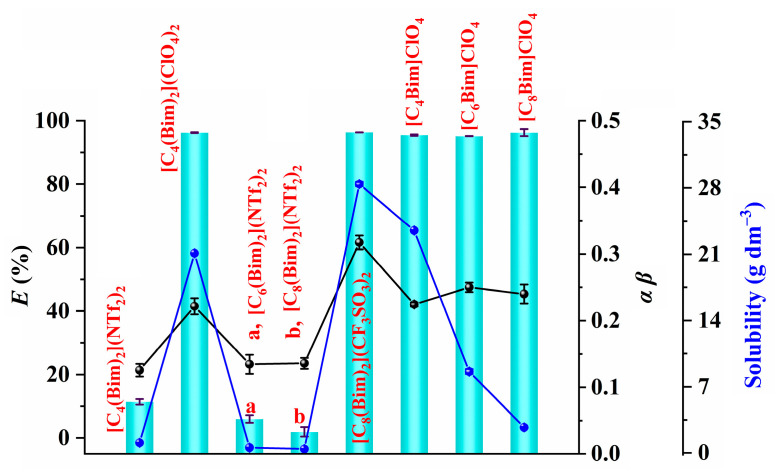
Correlation between the extraction ability of ILs and their hydrogen-bonding ability (black line) or water solubilities (blue line). Extraction conditions: 10 mg dm^−3^ of gold, 0.1 mol dm^−3^ of Na_2_S_2_O_3_, 1.0 mol dm^−3^ of NH_3_, 5 mmol dm^−3^ of CuSO_4_, 5:1 of volume ratio of water phase to IL phase (*V*_W_:*V*_IL_), 25 °C of extraction temperature, and 48 h of extraction time.

Figure 3a indicates that ILs with CF_3_SO_3_^−^ and ClO_4_^−^ anions have stronger extraction ability compared with those with the NTf_2_^−^ anion. Therefore, it is important to further investigate the driving forces behind this extraction. Previous studies have shown that the product of the hydrogen bond donating ability (*α*) and the hydrogen bond accepting ability (*β*) (*αβ*) can be utilized as a measure to assess the hydrogen-bonding strength of ILs [24]. This approach is also applied in the current study. As shown in Figure 4 (black line), the ILs containing the same anions exhibit similar levels of hydrogen-bonding strength, aligning with existing research [24]. Furthermore, ILs based on CF_3_SO_3_^−^- and ClO_4_^−^ exhibit higher extraction ability compared with those with NTf_2_^−^ as the anion. This suggests that ILs with strong hydrogen-bonding strength have higher extraction ability. This could be attributed to the weakening of electrostatic interaction between the IL cation and IL anion, caused by the hydrogen-bonding interaction between the IL and water. This weakening leads to an increase in the water solubilities of ILs (Figure 4, blue line) [25,26] and could potentially improve the electrostatic interaction between the IL cation and [Au(S_2_O_3_)_2_]^3−^, thereby enhancing the extraction ability of ILs.

In terms of [C_8_(Bim)_2_](CF_3_SO_3_)_2_, it exhibits higher hydrogen-bonding strength compared with ClO_4_^−^-based ILs, although its extraction ability is similar to the latter, which may be attributed to its higher water solubility (Figure 4, blue line). The higher water solubility leads to more significant dissolution loss of [C_8_(Bim)_2_](CF_3_SO_3_)_2_ during extraction, ultimately resulting in reduced extraction efficiency.

The above discussion suggests that the DILs [C_8_(Bim)_2_](CF_3_SO_3_)_2_ and [C_4_(Bim)_2_](ClO_4_)_2_ exhibit quicker extraction rates and greater extraction ability. Additionally, the latter has lower water solubility. Consequently, [C_4_(Bim)_2_](ClO_4_)_2_ was chosen as the optimal extractant and utilized in subsequent experiments.

### 2.2. Effect of Operating Parameters on the Extraction of [Au(S_2_O_3_)_2_]^3−^

**Figure 5:**
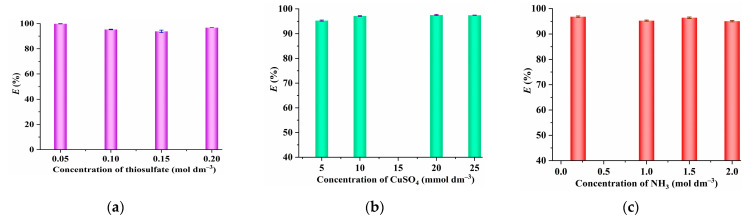
(**a**) The effect of thiosulfate concentration (5 mmol dm^−3^ of CuSO_4_; 1.0 mol dm^−3^ of NH_3_; extraction time, 20 min; extraction temperature, 25 °C; 10 mg dm^−3^ of gold; *V*_W_:*V*_IL_ = 5:1); (**b**) CuSO_4_ concentration (0.1 mol dm^−3^ of Na_2_S_2_O_3_; 1.0 mol dm^−3^ of NH_3_; extraction time, 20 min; extraction temperature, 25 °C; 10 mg dm^−3^ of gold; *V*_W_:*V*_IL_ = 5:1); and (**c**) NH_3_ concentration (0.1 mol Ldm^−3^ of Na_2_S_2_O_3_; 5 mmol dm^−3^ of CuSO_4_; extraction time, 20 min; extraction temperature, 25 °C; 10 mg dm^−3^ of gold; *V*_W_:*V*_IL_ = 5:1) on the extraction of [Au(S_2_O_3_)_2_]^3−^ by [C_4_(Bim)_2_](ClO_4_)_2_.

Generally, the leaching of gold typically involves the use of thiosulfate, CuSO_4_, and NH_3_ [1,2,4], and the effect of the thiosulfate concentration on the extraction of [Au(S_2_O_3_)_2_]^3−^ illustrated in Figure 5a indicates that the extraction efficiency of [C_4_(Bim)_2_](ClO_4_)_2_ toward [Au(S_2_O_3_)_2_]^3−^ almost remains constant with the thiosulfate concentration ranging from 0.05 mol dm^−3^ to 0.2 mol dm^−3^, suggesting that the extraction of [Au(S_2_O_3_)_2_]^3−^ by [C_4_(Bim)_2_](ClO_4_)_2_ can be carried out effectively over a broad spectrum of thiosulfate concentrations.. It has been proven that the presence of trace amounts of copper ions can improve the gold leaching rate, and the addition of NH_3_ is necessary to stabilize these copper ions [1,2,4]. The findings presented in Figure 5b show that the extraction efficiency of [Au(S_2_O_3_)_2_]^3−^ remains steady as the CuSO_4_ concentration varies from 5 mmol dm^−3^ to 25 mmol dm^−3^. Additionally, the introduction of NH_3_ does not affect the extraction efficiency of [Au(S_2_O_3_)_2_]^3−^, as shown in Figure 5c.

**Figure 6:**
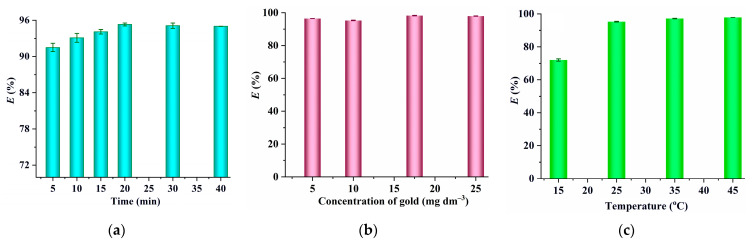
(**a**) The effect of extraction time (0.1 mol dm^−3^ of Na_2_S_2_O_3_; 5 mmol dm^−3^ of CuSO_4_; 1.0 mol dm^−3^ of NH_3_; extraction temperature, 25 °C; 10 mg dm^−3^ of gold; *V*_W_:*V*_IL_ = 5:1); (**b**) gold concentration (0.1 mol dm^−3^ of Na_2_S_2_O_3_; 5 mmol dm^−3^ of CuSO_4_; 1.0 mol dm^−3^ of NH_3_; extraction time, 20 min; extraction temperature, 25 °C; *V*_W_:*V*_IL_ = 5:1); and (**c**) extraction temperature (0.1 mol dm^−3^ of Na_2_S_2_O_3_; 5 mmol dm^−3^ of CuSO_4_; extraction time, 20 min; 1.0 mol dm^−3^ of NH_3_; 10 mg dm^−3^ of gold; *V*_W_:*V*_IL_ = 5:1) on the extraction of [Au(S_2_O_3_)_2_]^3−^ by [C_4_(Bim)_2_](ClO_4_)_2_.

The effect of extraction time on the extraction efficiency of [Au(S_2_O_3_)_2_]^3−^ is depicted in Figure 6a. It is evident that the extraction efficiency rises as the extraction time increases from 5 min to 20 min, after which it stabilizes with further extension of the extraction time. Consequently, 20 min was chosen for subsequent experiments. The results illustrated in Figure 6b suggest that the extraction efficiency of [Au(S_2_O_3_)_2_]^3−^ remains consistent as the gold concentration ranges from 5 mg dm^−3^ to 25 mg dm^−3^, highlighting the strong extraction ability of [C_4_(Bim)_2_](ClO_4_)_2_. Figure 6c indicates that the extraction efficiency of [Au(S_2_O_3_)_2_]^3−^ exceeds 95% in the temperature range of 25 °C to 45 °C but drops to 72.0% when the temperature is reduced to 15 °C. This suggests that the extraction of [Au(S_2_O_3_)_2_]^3−^ is sensitive to temperature and is best performed at room temperature or higher.

**Figure 7:**
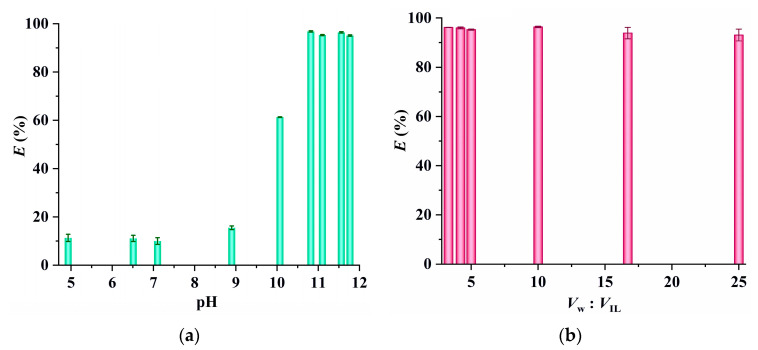
(**a**) The effect of equilibrium pH (0.1 mol dm^−3^ of Na_2_S_2_O_3_; 5 mmol dm^−3^ of CuSO_4_; extraction temperature, 25 °C; 10 mg dm^−3^ of gold; *V*_W_:*V*_IL_ = 5:1; extraction time, 20 min) and (**b**) phase volume ratio (0.1 mol dm^−3^ of Na_2_S_2_O_3_; 5 mmol dm^−3^ of CuSO_4_; extraction time, 20 min; 1.0 mol dm^−3^ of NH_3_; 10 mg dm^−3^ of gold; extraction temperature, 25 °C) on the extraction of [Au(S_2_O_3_)_2_]^3−^ by [C_4_(Bim)_2_](ClO_4_)_2_.

Additionally, the results illustrated in Figure 7a show that the extraction efficiency of [Au(S_2_O_3_)_2_]^3−^ remains constant within the pH range of 10.8 to 11.8 but significantly decreases as pH levels decrease, which may be ascribed to the fact that copper ions exist in the form of Cu(NH_3_)_4_^+^ in the presence of a high concentration of NH_3_. When pH values decrease, Cu(NH_3_)_4_^+^ converts to Cu(S_2_O_3_)_3_^5−^ due to the reaction between Cu^2+^ and S_2_O_3_^2−^ (Cu^2+^ + 4S_2_O_3_^2−^ = Cu(S_2_O_3_)_3_^5−^ + 0.5S_4_O_6_^2−^) [11,27]. This copper–thiosulphate complex can also be extracted by [C_4_(Bim)_2_](ClO_4_)_2_, leading to competition with the extraction of [Au(S_2_O_3_)_2_]^3−^ and consequently reducing the extraction efficiency of [Au(S_2_O_3_)_2_]^3−^. To confirm this hypothesis, the extraction efficiencies of Cu^2+^ (5 mmol dm^−3^) at pH 11, pH 7, and pH 4.5 were measured. It is observed that the extraction efficiency of Cu^2+^ at pH 11 is zero, while at pH 7 and pH 4.5, it increases to 11.7% and 14.6% respectively, supporting the above deduction. Finally, as shown in Figure 7b, the extraction efficiency of [Au(S_2_O_3_)_2_]^3−^ remains above 93% across a wide range of phase volume ratios (*V*_w_:*V*_IL_, 3.3 to 25). A phase volume ratio of 10 was chosen for subsequent experiments due to its ability to achieve an extraction efficiency as high as 96.4%.

### 2.3. Extraction Mechanism

Recently, Mahandra and coworkers have reported that the extraction of [Au(S_2_O_3_)_2_]^3−^ by a phosphonium-based IL–toluene system is an anion exchange process [10,11]. If the extraction of [Au(S_2_O_3_)_2_]^3−^ by [C_4_(Bim)_2_](ClO_4_)_2_ also operates through an anion exchange mechanism, the extraction process can be described as follows:

3{[C_4_(Bim)_2_](ClO_4_)_2_}_IL_ + 2{[Au(S_2_O_3_)_2_]^3−^}_W_ ⇋ {[C_4_(Bim)_2_]_3_[Au(S_2_O_3_)_2_]_2_}_IL_ + 6(ClO_4_^−^)_W_ (1)

where the subscripts IL and W refer to the IL and water phases, respectively.

**Figure 8:**
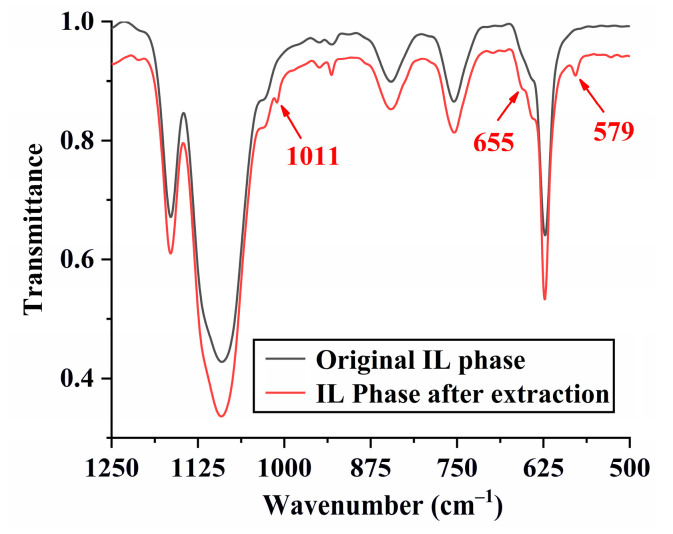
The FT-IR spectra of the IL phases before and after extraction.

As listed in Equation (1), the anion exchange mechanism means an increase in the concentration of ClO_4_^−^ after extraction. Further experiments were thus carried out to confirm this hypothesis by extracting 7.5 × 10^−3^ mol dm^−3^ of [Au(S_2_O_3_)_2_]^3−^ with [C_4_(Bim)_2_](ClO_4_)_2_. A high extraction efficiency of 99.1% means that the increment of ClO_4_^−^ concentration in water post-extraction should be 2.23 × 10^−2^ mol dm^−3^, which closely aligns with the experimental value (2.31 × 10^−2^ mol dm^−3^) determined through ion chromatography. This result suggests that the extraction of [Au(S_2_O_3_)_2_]^3−^ with [C_4_(Bim)_2_](ClO_4_)_2_ is an anion exchange process. Additionally, the FT-IR spectra of the IL phases pre- and post-extraction, as demonstrated in Figure 8, reveal the emergence of three new peaks at 1011 cm^−1^, 655 cm^−1^, and 579 cm^−1^ in the IR spectrum of the IL phase after extraction. These peaks correspond to the symmetrical stretching of S=O, S-O stretching, and S-S stretching vibrations, respectively [28,29], indicating the transfer of [Au(S_2_O_3_)_2_]^3−^ from the water phase to the IL phase.

### 2.4. Thermodynamic Analysis

To delve deeper into how extraction works, a thermodynamic analysis was conducted to determine the Gibbs free-energy change (Δ*G*), enthalpy change (Δ*H*), and the entropy change (Δ*S*) of the extraction process. This analysis was based on equations that assume Δ*H* and Δ*S* remain consistent across the temperature range investigated [30,31].

Δ*G* = −R*T*ln*D* (2)

where R and *T* are the gas constant and the extraction temperature, respectively.

ln*D* = −Δ*H*/R*T*+Δ*S*/R (3)

The van’t Hoff plot for the extraction of [Au(S_2_O_3_)_2_]^3−^ is shown in Figure S1 in the Supplementary Materials, with the resulting thermodynamic parameters detailed in Table 1. Negative Δ*G* values signify the spontaneity of the [Au(S_2_O_3_)_2_]^3−^ extraction process. A positive Δ*H* indicates that the extraction is endothermic, while a positive Δ*S* suggests increased disorder at the liquid–liquid interface during the extraction [30,31].

### 2.5. Comparison of [C_4_(Bim)_2_](ClO_4_)_2_ with the Reported Extraction Solvents

**Figure 9:**
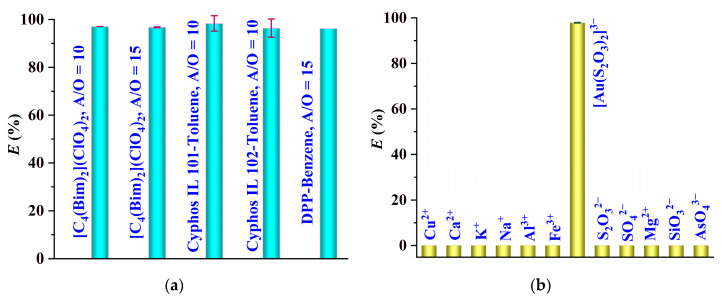
(**a**) Comparison of [C_4_(Bim)_2_](ClO_4_)_2_ with the reported extraction systems (A/O, the volume ratio of water phase to organic phase; 0.2 mol dm^−3^ of Na_2_S_2_O_3_; 10 mg dm^−3^ of gold); (**b**) extraction behavior of [C_4_(Bim)_2_](ClO_4_)_2_ toward [Au(S_2_O_3_)_2_]^3−^ and coexisting ions.

Previously, Cyphos IL 101–toluene, Cyphos IL 102–toluene [10,11], and DPP–benzene [9] were applied to the recovery of [Au(S_2_O_3_)_2_]^3−^ from thiosulfate solutions, and the results shown in Figure 9a indicate that the extraction efficiencies of [C_4_(Bim)_2_](ClO_4_)_2_ toward [Au(S_2_O_3_)_2_]^3−^ are comparable to those of Cyphos IL 101 (102)–toluene and DPP–benzene systems. This suggests that [C_4_(Bim)_2_](ClO_4_)_2_ has a strong ability to extract [Au(S_2_O_3_)_2_]^3−^ and could be a more environmentally friendly solvent option due to its lack of toxic and volatile components. Additionally, the gold extracted into the IL phase can be recovered through back extraction using an ethylenediaminetetraacetic acid disodium aqueous solution (0.1 mol dm^−3^) as the extraction solvent, with a high back-extraction efficiency of 94.4%. The recovered IL phase can be reused for subsequent extraction cycles without significantly decreasing its extraction ability (96.9% for recovered IL compared to 97.9% for fresh IL). Furthermore, the structure of the recovered IL was examined by proton nuclear magnetic resonance (^1^H NMR) spectroscopy, and the results depicted in Figure S2 indicate that the ^1^H NMR spectrum of the recovered [C_4_(Bim)_2_](ClO_4_)_2_ is the same as that of the initial one, indicating that [C_4_(Bim)_2_](ClO_4_)_2_ maintains its original chemical structure even after being used.

### 2.6. Selective Recovery of Gold from Real Samples

To assess the potential of using [C_4_(Bim)_2_](ClO_4_)_2_ to isolate [Au(S_2_O_3_)_2_]^3−^ from the actual thiosulfate leaching solution of a gold ore with a gold concentration of 6.0 mg dm^–1^, extraction experiments were carried out at a phase volume ratio (*V*_W_:*V*_IL_) of 10, and the results illustrated in Figure 9b indicate that the extraction efficiency of [Au(S_2_O_3_)_2_]^3−^ closely resembles that of the simulated thiosulfate leaching solution (Figure 4). This suggests that the presence of other ions may not impede the extraction of [Au(S_2_O_3_)_2_]^3−^, as confirmed by interference tests (Figure 9b, where the extraction efficiencies of the coexisting ions are zero). Therefore, it can be inferred that [C_4_(Bim)_2_](ClO_4_)_2_ exhibits high selectivity for the extraction of [Au(S_2_O_3_)_2_]^3−^.

## 3. Materials and Methods

### 3.1. Reagents and Materials

1-(n-Butyl)imidazole (Bim, 98%), 1,4-dibromobutane (98%), 1,6-dibromohexane (97%), 1,8-dibromooctane (98%), n-butylbromide (>99%), barium perchlorate hydrate (Ba(ClO_4_)_2_·3H_2_O, 99%), potassium trifluoromethanesulfonate (KCF_3_SO_3_, 98%), bis(trifluoromethane)sulfonimide lithium (LiNTf_2_, 99.9%), and gold sodium thiosulfate (Na_3_Au(S_2_O_3_)_2_.xH_2_O, 99.9%) were supplied by Macklin Biochemical Technology Co., Ltd. (Shanghai, China). The ILs 1-butyl-3-hexylimidazolium perchlorate ([C_6_Bim]ClO_4_) and 1-butyl-3-octylimidazolium perchlorate ([C_8_Bim]ClO_4_) were prepared as described in our previous work [32]. Reichardt’s dye (RD, 90%) and 4-nitroaniline (NA, ≥99%) were obtained from Sigma-Aldrich Co.; *N*,*N*-diethyl-4-nitroaniline (DENA, 97%) was purchased from Fluorochem Ltd., (Hadfield, UK). Unless stated otherwise, all other reagents are analytical grade. Deionized water was used throughout the experiments. Dried gold ore powder (particle size, around 200 mesh; gold content, 60 g t^−1^) was obtained from a Xinjiang Gold Mine Company (Xinjiang, China), and its main components, measured by an X-ray fluorescence spectrometer (XRF, ARL Perform’X, Thermo Fisher Scientific Inc., Waltham, CA, USA), are 29.8% of Ca, 23.2% of Fe, 22.4% of Si, 7.5% of S, 6.0% of Al, 3.2% of As, 2.2% of Mg, 2.0% of K, 1.3% of Na, and 1.3% of Ti. The leaching of gold from gold ore was performed as follows [33,34]: First, 20 g of gold ore was left to contact with 100 mL of lixiviant composed of 0.1 mol dm^−3^ of Na_2_S_2_O_3_, 5.0 mmol dm^−3^ of CuSO_4_, and 1.0 mol dm^−3^ of NH_3_ under stirring at room temperature for 48 h.

### 3.2. Synthesis of Dicationic Imidazolium-Based ILs

1,4-Bis[*N*-(*N*’-butylimidazolium)]butane perchlorate ([C_4_(Bim)_2_](ClO_4_)_2_) was prepared as follows: First, 0.06 mol of 1,4-dibromobutane and 0.12 mol of Bim were mixed under stirring at 70 °C for 7 h, and the resultant product was dissolved by 40 mL of water, followed by the addition of 0.12 mol of BaClO_4_ to generate the IL [C_4_(Bim)_2_](ClO_4_)_2_. After being washed by water several times until Br-free, as indicated by a AgNO_3_ test of the water washings [35], the IL phase was dried at 70 °C, and [C_4_(Bim)_2_](ClO_4_)_2_ was obtained as a light-yellow liquid (yield, 73.0%).

The synthesis of the ILs [C_4_(Bim)_2_](NTf_2_)_2_ (light-yellow liquid, 97.6% yield) and [C_4_(Bim)_2_](CF_3_SO_3_)_2_ (white solid, melting point: 54.8–57.7 °C; 7.0% yield) followed the same procedure as for [C_4_(Bim)_2_](ClO_4_)_2_ described above, although LiNTf_2_ or KCF_3_SO_3_ was used instead of Ba(ClO_4_)_2_.

1,4-Bis[*N*-(*N*’-butylimidazolium)]hexane-based ([C_6_(Bim)_2_](ClO_4_)_2_ (white solid, melting point: 46.4–48.1 °C; 79.9% yield), [C_6_(Bim)_2_](NTf_2_)_2_ (light-yellow liquid, 97.9% yield), and [C_6_(Bim)_2_](CF_3_SO_3_)_2_ (white solid, melting point: 55.8–56.4 °C; 22.8% yield)); 1,4-bis[*N*-(*N*’-butylimidazolium)]octane-based ([C_8_(Bim)_2_](ClO_4_)_2_ (white solid, melting point: 85.7–87.1 °C; 79.1% yield), [C_8_(Bim)_2_](NTf_2_)_2_ (light-yellow liquid, 92.3% yield), and [C_8_(Bim)_2_](CF_3_SO_3_)_2_ (light-yellow liquid, 46.3% yield)); and 1-butyl-3-butylimidazolium-based ([C_4_Bim]ClO_4_ (light-yellow liquid, 69.0% yield)) ILs were prepared via the same procedure as for [C_4_(Bim)_2_]^2+^-based ILs described above. All the synthesized ILs were characterized by ^1^H NMR (model Ascend 400, 400 MHz, Bruker Biospin, Rheinstetten, Germany), and the corresponding spectra are demonstrated in Figures S3–S12 in the Supplementary Materials.

### 3.3. Measurements of the Solubility, Viscosity, and Conductivity

The solubilities of ILs were determined via a previously reported method [36] as follows: The excess amount of a specific IL was mixed with water under stirring for 4 h at 25 °C, and the resultant mixture was left to rest for 12 h to achieve complete phase separation. The IL concentration (solubility) in the water phase was measured by an ultraviolet–visible (UV–Vis) spectrophotometer (Purkinje General Instrument Co., Beijing, China) at 220 nm. The selection of 220 nm as the detection wavelength is based on the characteristics of their ultraviolet absorption spectra, as shown in Figures S13–S16 in the Supplementary Materials [37].

The viscosities of IL phases saturated with water and the water phases saturated with ILs were determined at 25 °C using an NDJ-8S rotary viscometer at a rotation speed of 60 rpm per minute (Changji Geological Instrument Co., Ltd., Shanghai, China). The relative standard deviations of the test viscosity values were in the range of 0.08% to 4.5%.

The conductivities of IL phases saturated with water and the water phases saturated with ILs were determined at 25 °C with a conductivity meter (model SLDS, Sangli Electronic Equipment Factory, Nanjing, China).

### 3.4. Measurements of Hydrogen Bond Donating Ability and Hydrogen Bond Accepting Ability

The hydrogen-bond donating ability (*α*) and the hydrogen-bond accepting ability (*β*) of the ILs used in this work were determined by using the three dyes, RD, DENA, and NA as probes via a previously reported method [38]. Detailed information is listed in the Supplementary Materials.

### 3.5. Calculation of the Electrophilicity of ILs

The electrophilicity of ILs was quantitatively expressed by the electrophilicity index and calculated using the Multiwfn software (version 3.8) [39].

### 3.6. Extraction Procedure

For a typical extraction procedure, 1.0 mL of a specific IL was mixed with 5.0 mL of [Au(S_2_O_3_)_2_]^3−^ solution (10 mg dm^−3^ of gold, 0.1 mol dm^−3^ of Na_2_S_2_O_3_, 1.0 mol dm^−3^ of NH_3_, and 5 mmol dm^−3^ of CuSO_4_) under stirring at 25 °C for 20 min. After centrifugation, the gold concentration in the water phase was determined by an atomic absorption spectrophotometer (AAS, TAS-990, Beijing Puxi General Instrument Co., Ltd., Beijing, China) [6,7,8,9,10,11]. The elemental contents of the actual thiosulfate gold leaching solution were measured by inductively coupled plasma optical emission spectrometry (ICP-OES, model Optima 8000, Perkin Elmer, Waltham, MA, USA) [6,7,8,9,10,11]. Ammonia solutions ranging from 0.2 mol dm^−3^ to 2 mol dm^−3^ were employed to control the aqueous pH in the range of 10 to 11.8, and 0.1 mol dm^−3^ of ammonium buffer solution was utilized to keep the pH within the range of 8 to 10, while 0.1 mol dm^−3^ of acetic acid solution was used to regulate the aqueous pH under acidic or neutral conditions.

The extraction efficiency (*E*), the distribution ratio (*D*), and the relationship between *E* and *D* can be calculated using the following equations:

*E*% = (1 − *C*_w_/*C*_w_°) × 100 (4)

*D* = *C*_w_°/*C*_w_ = *E* × (*V*_w_/*V*_IL_)/(1 − *E*) (5)

where C_w_° and C_w_ are the concentrations of gold in the water phase before and after extraction, respectively. The symbols V_w_ and V_IL_ refer to the volumes of water and IL phases, respectively.

### 3.7. Ion Chromatographic (IC) Analysis

The IC analysis was conducted on an ion chromatograph (2100 model, Thermo Fisher Scientific Inc., Waltham, CA, USA) equipped with a conductivity detector, and the chromatographic conditions were as follows: separation column, IonPac AS 11-HC (250 mm × 4 mm); guard column, IonPac AG11-HC (50 mm × 4 mm); eluent, 20 mmol dm^−3^ NaOH with a flow rate of 1.0 mL min^−1^; suppression current, 60 mA; column temperature, 35 °C; injection volume, 15 µL.

The Fourier transform infrared (FT-IR) spectra of the IL phase before and after extraction were measured using a Frontier FT-IR spectrophotometer (PerkinElmer Inc., Waltham, MA, USA).

All the experiments were carried out in triplicate, and the results were expressed as mean ± standard deviation (SD).

## 4. Conclusions

In this work, a series of DILs were prepared and used to extract gold from a thiosulfate leaching solution. The experimental results suggested that the ILs with strong hydrogen-bonding ability exhibited high extraction ability toward [Au(S_2_O_3_)_2_]^3−^. The extraction efficiency of [Au(S_2_O_3_)_2_]^3−^ by DILs was significantly affected by the aqueous pH, as there was a competitive extraction between Cu(S_2_O_3_)_3_^5−^ and [Au(S_2_O_3_)_2_]^3−^ at pH levels ≤ 10, consequently reducing the extraction ability of ILs toward [Au(S_2_O_3_)_2_]^3−^. The extraction of [Au(S_2_O_3_)_2_]^3−^ by DILs followed an anion exchange mechanism, was endothermic, and involved increased entropy. Anion exchange at the IL–water interface was identified as the rate-limiting step in the extraction process. The presence of other ions in the thiosulfate leaching solution did not hinder the extraction of [Au(S_2_O_3_)_2_]^3−^, and gold could be recovered from the IL phase through back extraction. The DILs exhibited superior extraction ability compared with traditional liquid–liquid extraction systems, suggesting their potential as environmentally friendly alternatives to volatile and hazardous solvents for gold recovery from thiosulfate leaching solutions.

## Figures and Tables

**Table 1:** Thermodynamic parameters for the extraction of [Au(S_2_O_3_)_2_]^3−^ by [C_4_(Bim)_2_](ClO_4_)_2_.

*T* (K)	Δ*G* (kJ mol^−1^)	Δ*H* (kJ mol^−1^)	Δ*S* (J mol^−1^ K^−1^)
288	−6.4	72.7	278
298	−11.7		
308	−13.4		
318	−14.9		

## Data Availability

The data presented in this study are available in article and supplementary materials.

## Supplementary Materials

The following supporting information can be downloaded at: https://www.mdpi.com/article/10.3390/molecules29112659/s1, Figure S1: The van’t Hoff plot for the extraction of [Au(S_2_O_3_)_2_]^3−^; Figure S2: The ^1^H NMR spectra of the fresh and the recovered [C_4_Bim]ClO_4_ (400 MHz, DMSO-*d*_6_); Figures S3–S12: the ^1^H NMR spectra of [C_4_(Bim)_2_](ClO_4_)_2_, [C_4_(Bim)_2_](CF_3_SO_3_)_2_, [C_4_(Bim)_2_](NTf_2_)_2_, [C_6_(Bim)_2_](ClO_4_)_2_, [C_6_(Bim)_2_](CF_3_SO_3_)_2_, [C_6_(Bim)_2_](NTf_2_)_2_, [C_8_(Bim)_2_](CF_3_SO_3_)_2_, [C_8_(Bim)_2_](NTf_2_)_2_, [C_8_(Bim)_2_](ClO_4_)_2_, [C_4_Bim]ClO_4_; Figures S13–S16: Ultraviolet absorption spectra of [C_4_(Bim)_2_](ClO_4_)_2_, [C_6_(Bim)_2_](ClO_4_)_2_, [C_8_(Bim)_2_](ClO_4_)_2_, [C_4_(Bim)_2_](NTf_2_)_2_, [C_6_(Bim)_2_](NTf_2_)_2_, [C_8_(Bim)_2_](NTf_2_)_2_, [C_8_(Bim)_2_](CF_3_SO_3_)_2_, [C_4_(Bim)_2_](ClO_4_)_2_; measurements of hydrogen-bond donating ability and hydrogen-bond accepting ability of the ILs.
