# Supplementary material for: Highly Efficient and Selective Extraction of Gold from Thiosulfate Leaching Solution Using Functionalized Dicationic Ionic Liquids
Source: Molecules. 2024 Jun 4;29(11):2659. doi: 10.3390/molecules29112659 (PMC11174032; doi:10.3390/molecules29112659)
Supplement: Supplementary file 1 [file molecules-29-02659-s001.zip › molecules-3003799-supplementary.pdf]

Highly efficient and selective extraction of gold from thiosulfate leaching solution using functionalized dicationic ionic liquids

Qiang Zhou [1], Yunchang Fan [1,*] and Sheli Zhang [2]

[1]College of Chemistry and Chemical Engineering, Henan Polytechnic University, Jiaozuo 454003, China; 212112010027@home. hpu.edu.cn (Q.Z.); fanyunchang@hpu.edu.cn (Y.F.)

[2]School of Science and Technology, Jiaozuo Teachers College, Jiaozuo 454000, China; 1295006035@jzsz.edu.cn (S.Z.)

*Corresponding author. E-mail address: fanyunchang@hpu.edu.cn

Tel.: +863913986813, Fax: +863913987815.

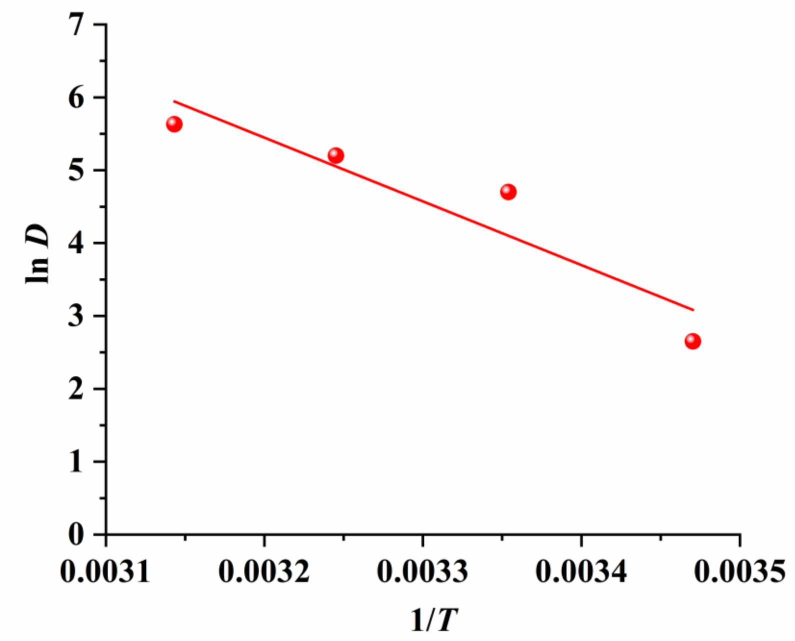

Figure S1. Van't Hoff's plot for the extraction of $Au(S_2O_3)_2^{3-}$ by $[C_4(Bim)_2](ClO_4)_2$.

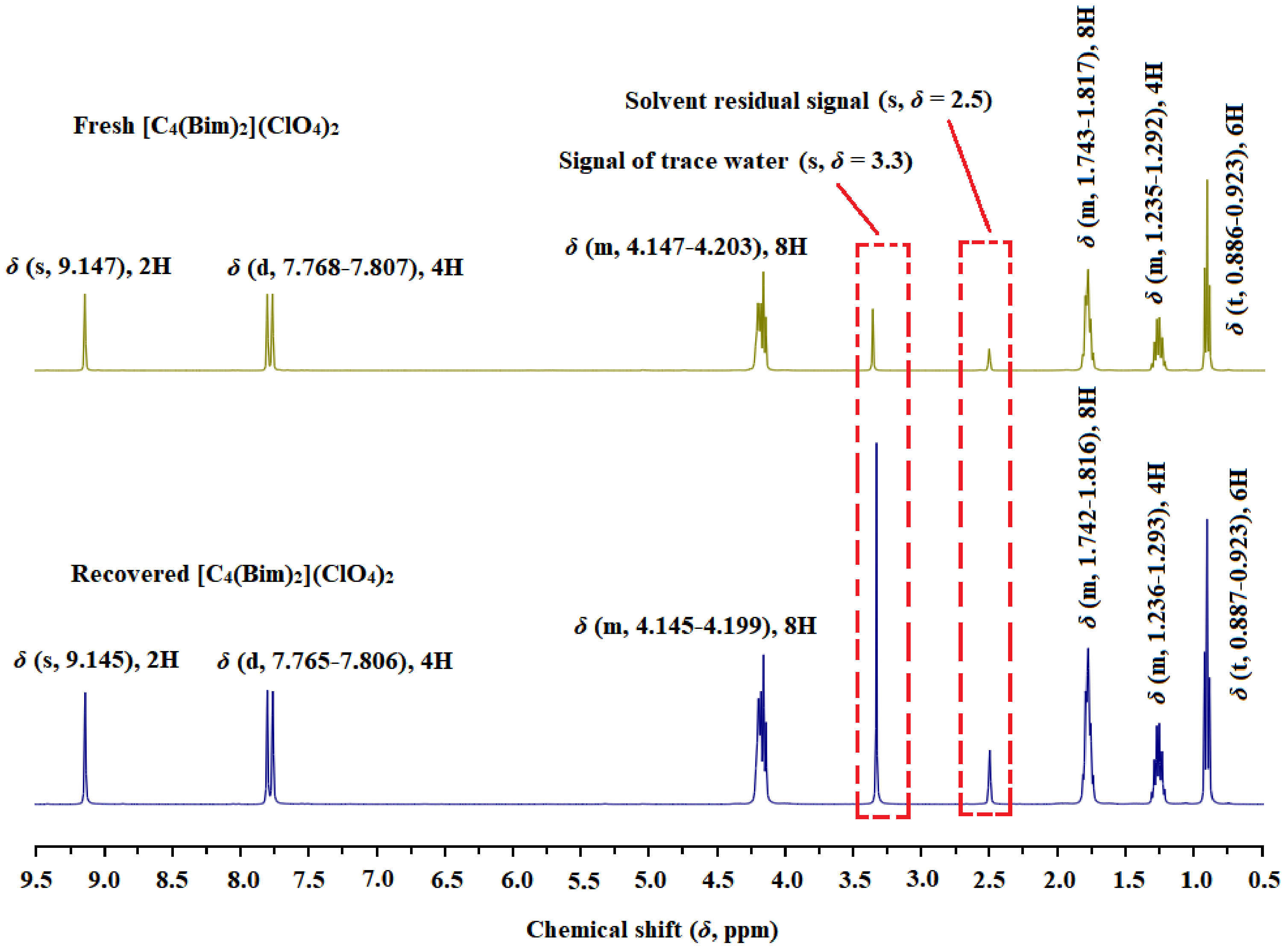

Figure S2. The ^{1}H NMR spectra of the fresh and the recovered [C_4Bim]ClO_4 (400 MHz, DMSO-d_6).

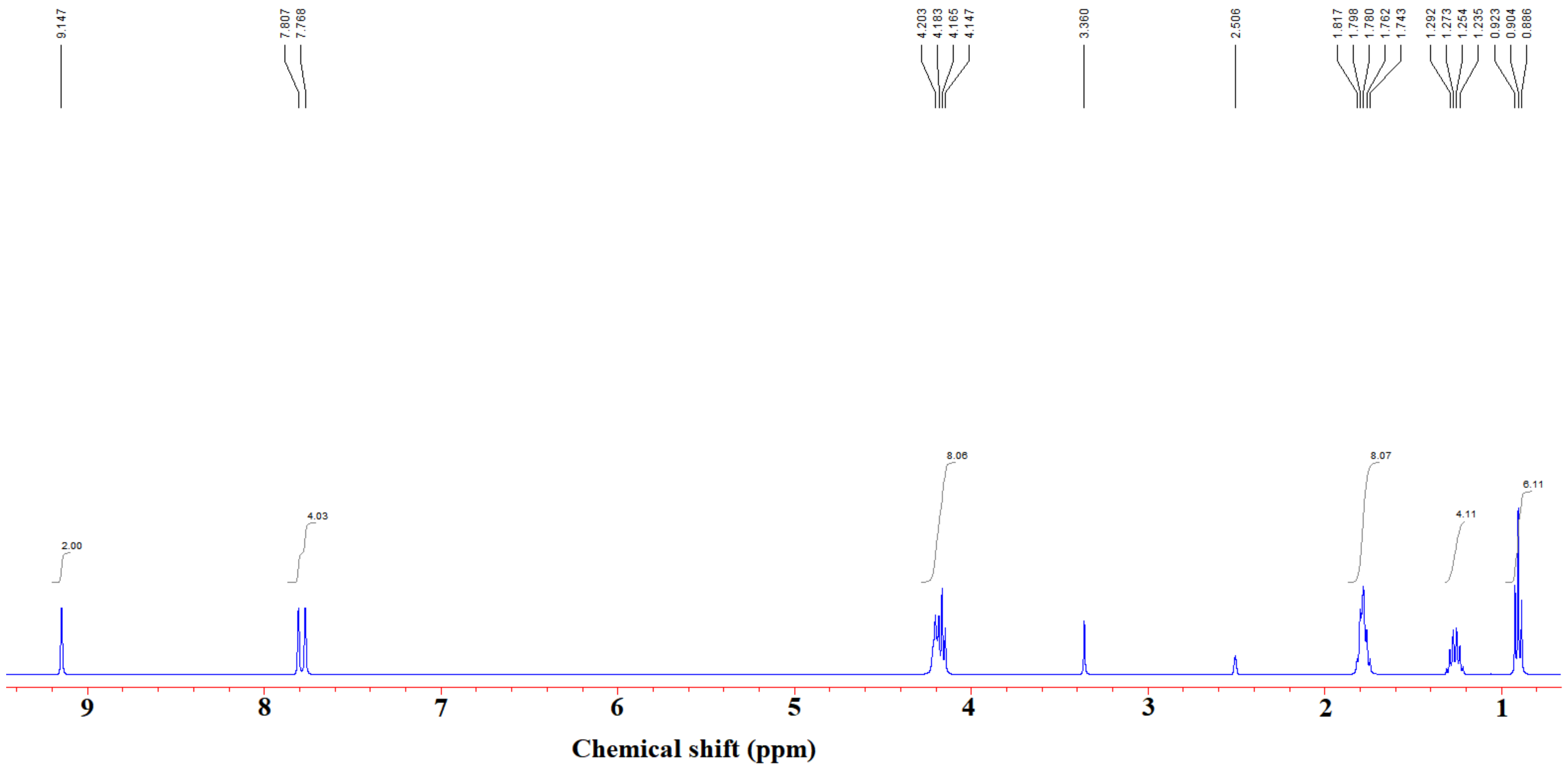

Figure S3. The ^{1}H NMR spectrum of $[C_4(Bim)_2](ClO_4)_2$ (400 MHz, DMSO-d_6).

Chemical shifts (ppm): 0.886-0.923 (t, 6H), 1.235-1.292 (m, 4H), 1.743-1.817 (m, 8H), 4.147-4.203 (m, 8H), 7.768-7.807 (d, 4H), 9.147 (s, 2H).

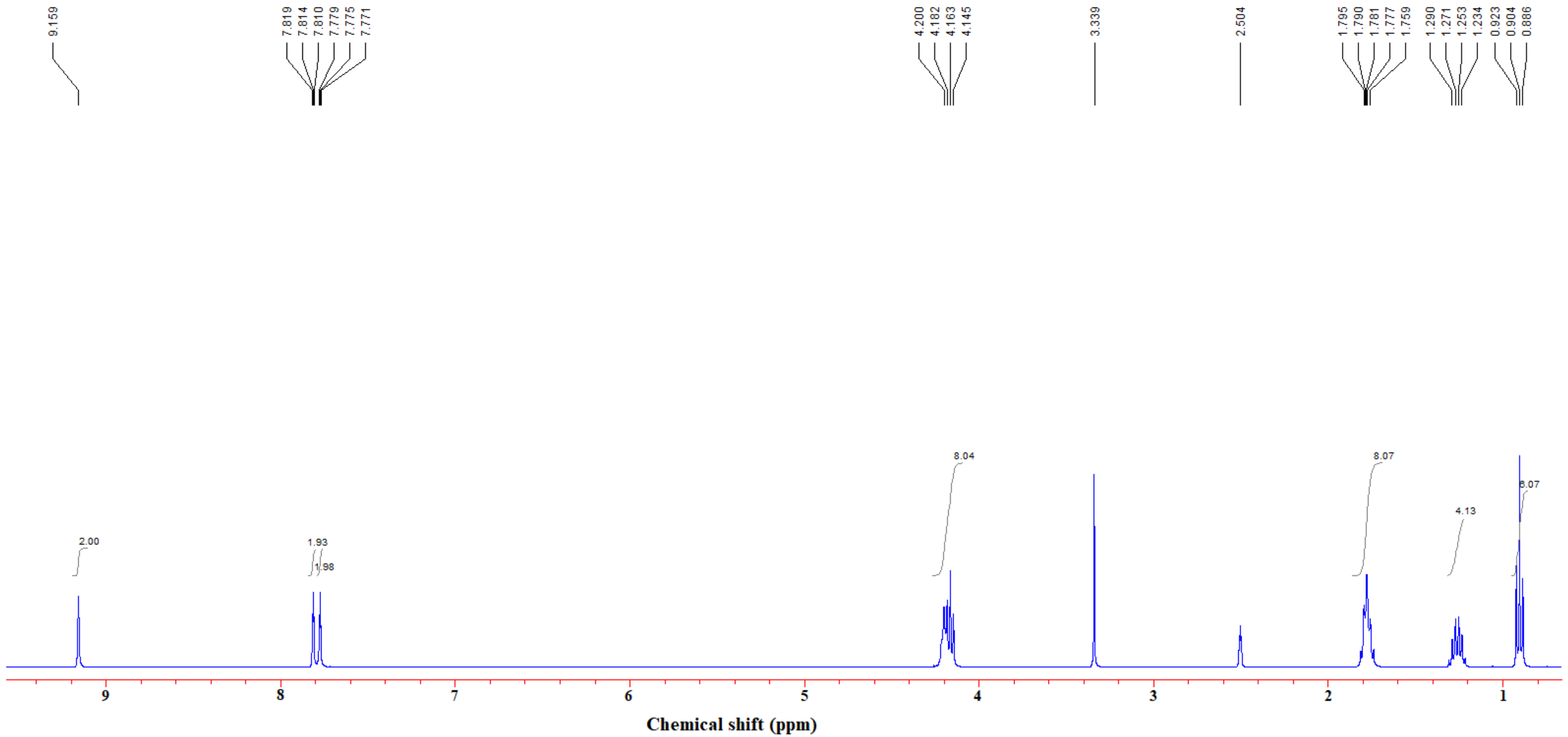

Figure S4. The ^{1}H NMR spectrum of $[C_4(Bim)_2](CF_3SO_3)_2$ (400 MHz, DMSO-d_6).

Chemical shift (ppm): 0.886-0.923 (t, 6H), 1.234-1.290 (m, 4H), 1.759-1.795 (m, 8H), 4.145-4.200 (m, 8H), 7.771-7.779 (t, 2H), 7.810-7.819 (t, 2H), 9.159 (s, 2H).

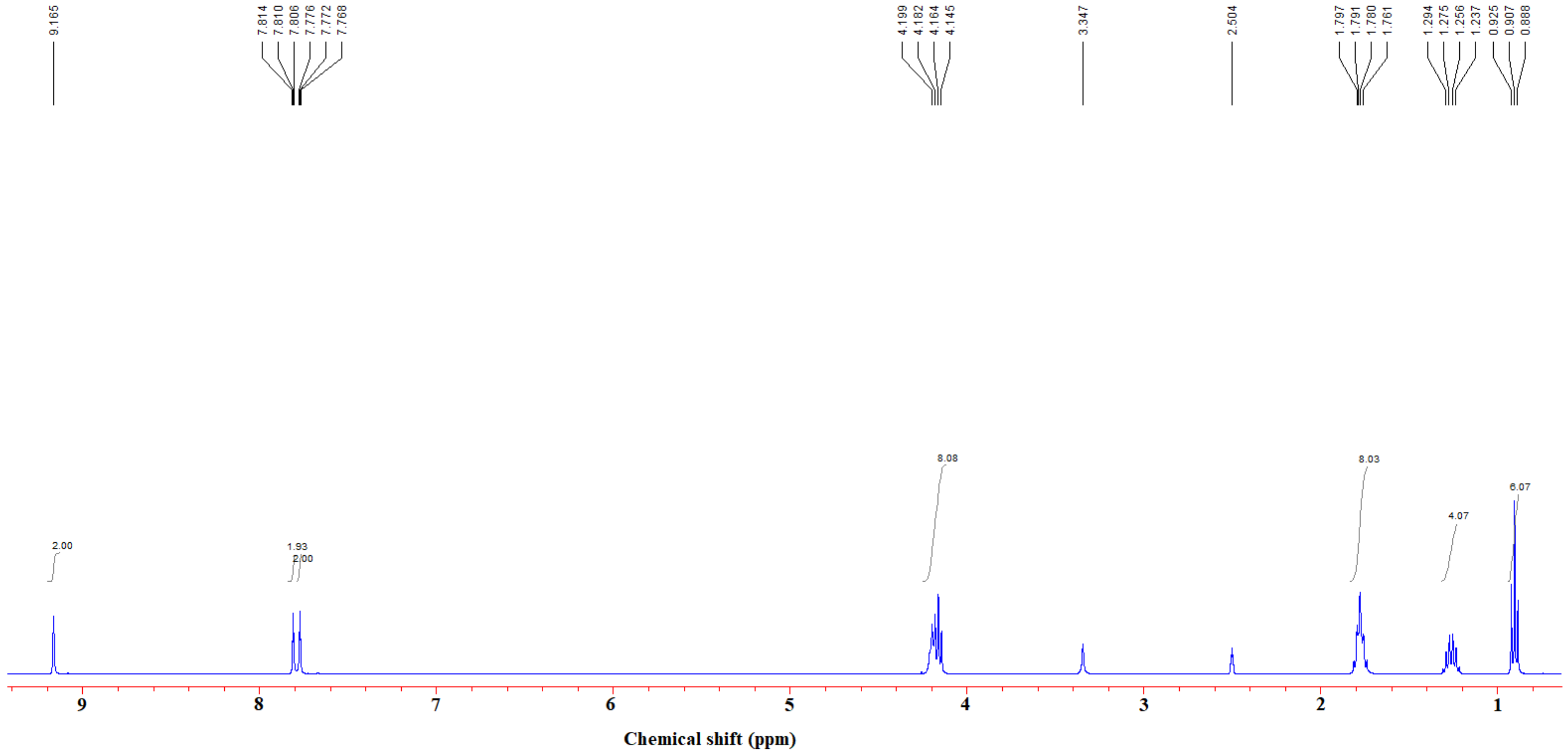

Figure S5. The ^{1}H NMR spectrum of $[C_4(Bim)_2](NTf_2)_2$ (400 MHz, DMSO-d_6).

Chemical shift (ppm): 0.888-0.925 (t, 6H), 1.237-1.294 (m, 4H), 1.761-1.797 (m, 8H), 4.145-4.199 (m, 8H), 7.768-7.776 (t, 2H), 7.806-7.814 (t, 2H), 9.165 (s, 2H)

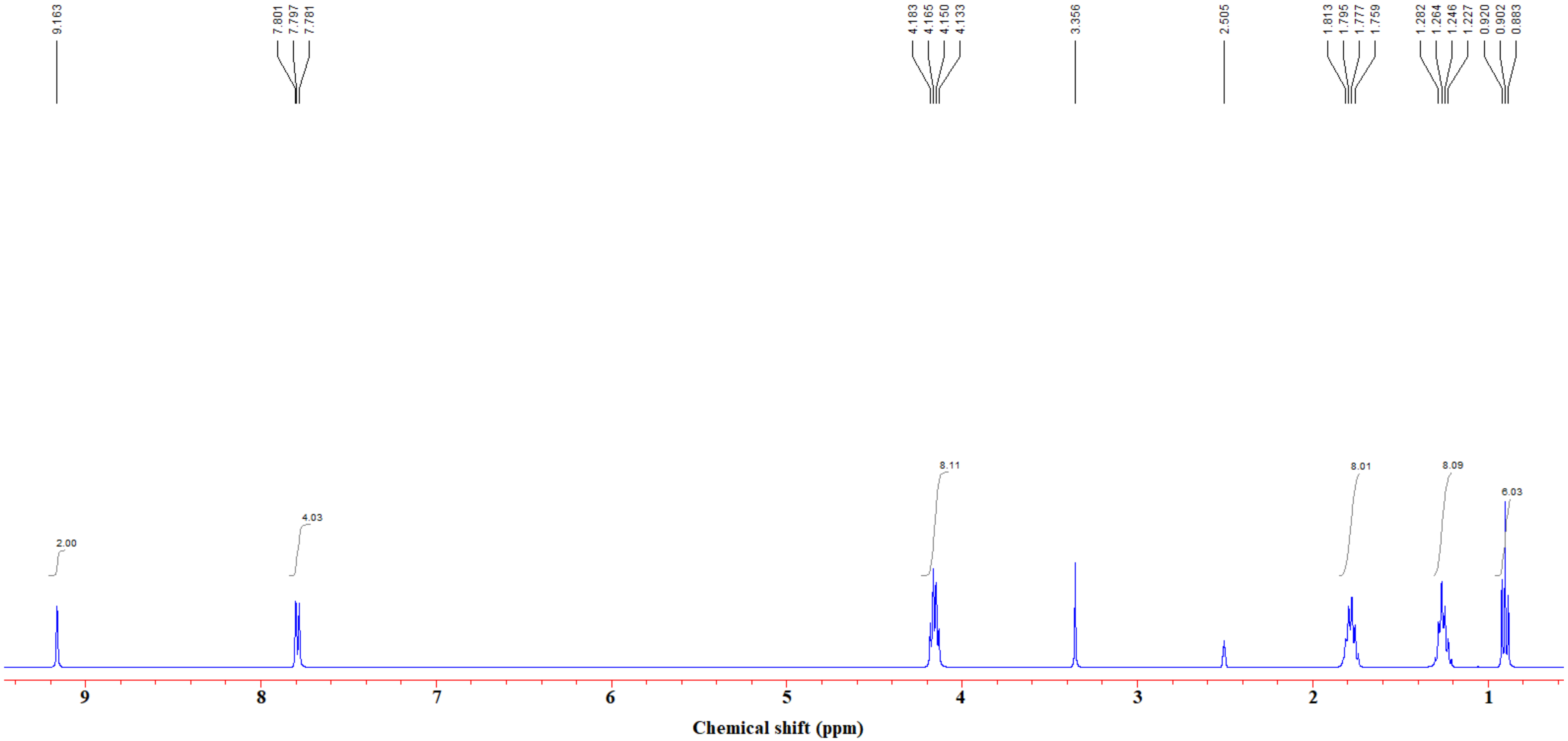

Figure S6. The ^{1}H NMR spectrum of $[C_6(Bim)_2](ClO_4)_2$ (400 MHz, DMSO-d_6).

Chemical shift (ppm): 0.883-0.920 (t, 6H), 1.227-1.282 (m, 8H), 1.759-1.813 (m, 8H), 4.133-4.183 (m, 8H), 7.781-7.801 (t, 4H), 9.163 (s, 2H).

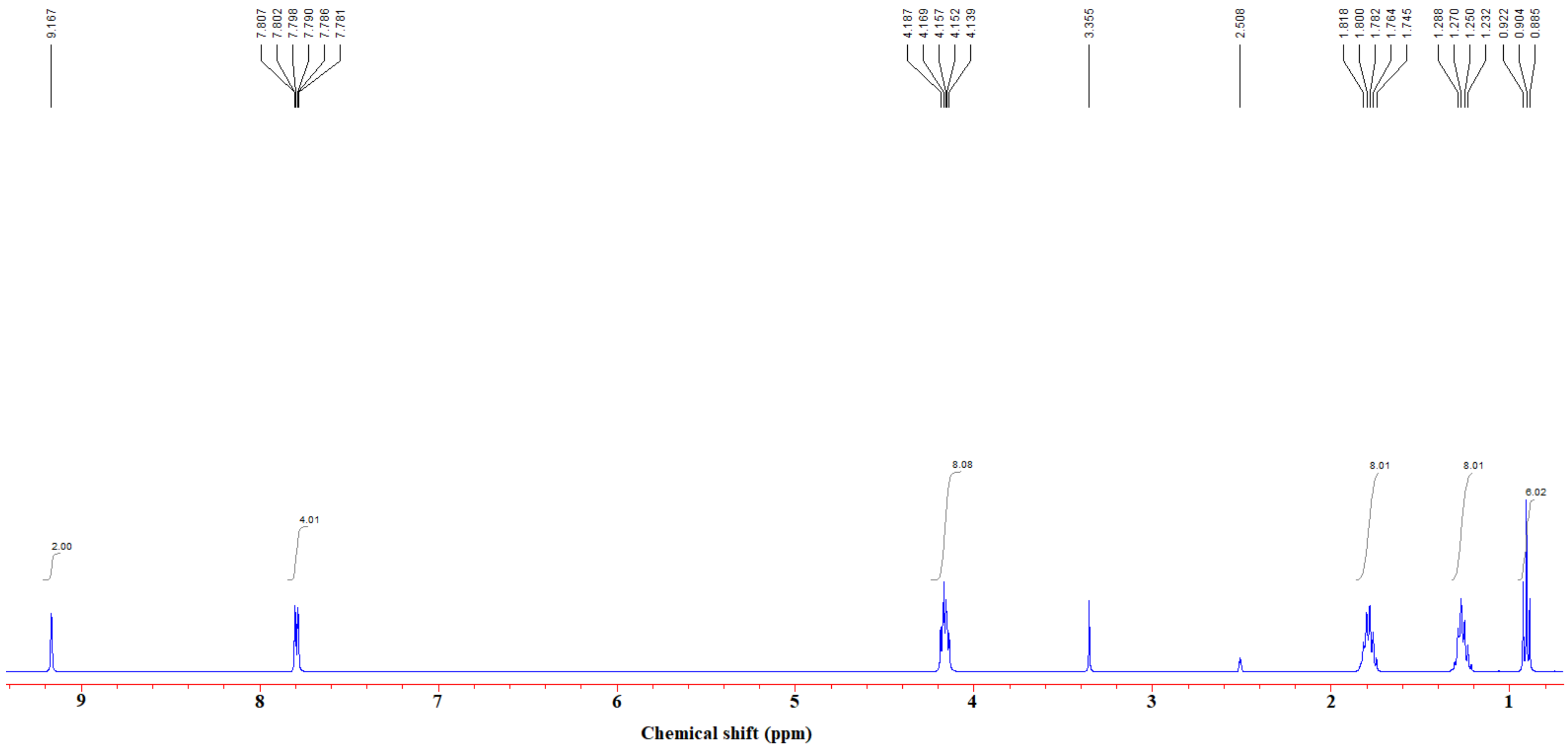

Figure S7. The ^{1}H NMR spectrum of $[C_6(Bim)_2](CF_3SO_3)_2$ (400 MHz, DMSO-d_6).

Chemical shift (ppm): 0.885-0.922 (t, 6H), 1.232-1.288 (m, 8H), 1.745-1.818 (m, 8H), 4.139-4.187 (m, 8H), 7.781-7.807 (m, 4H), 9.167 (s, 2H).

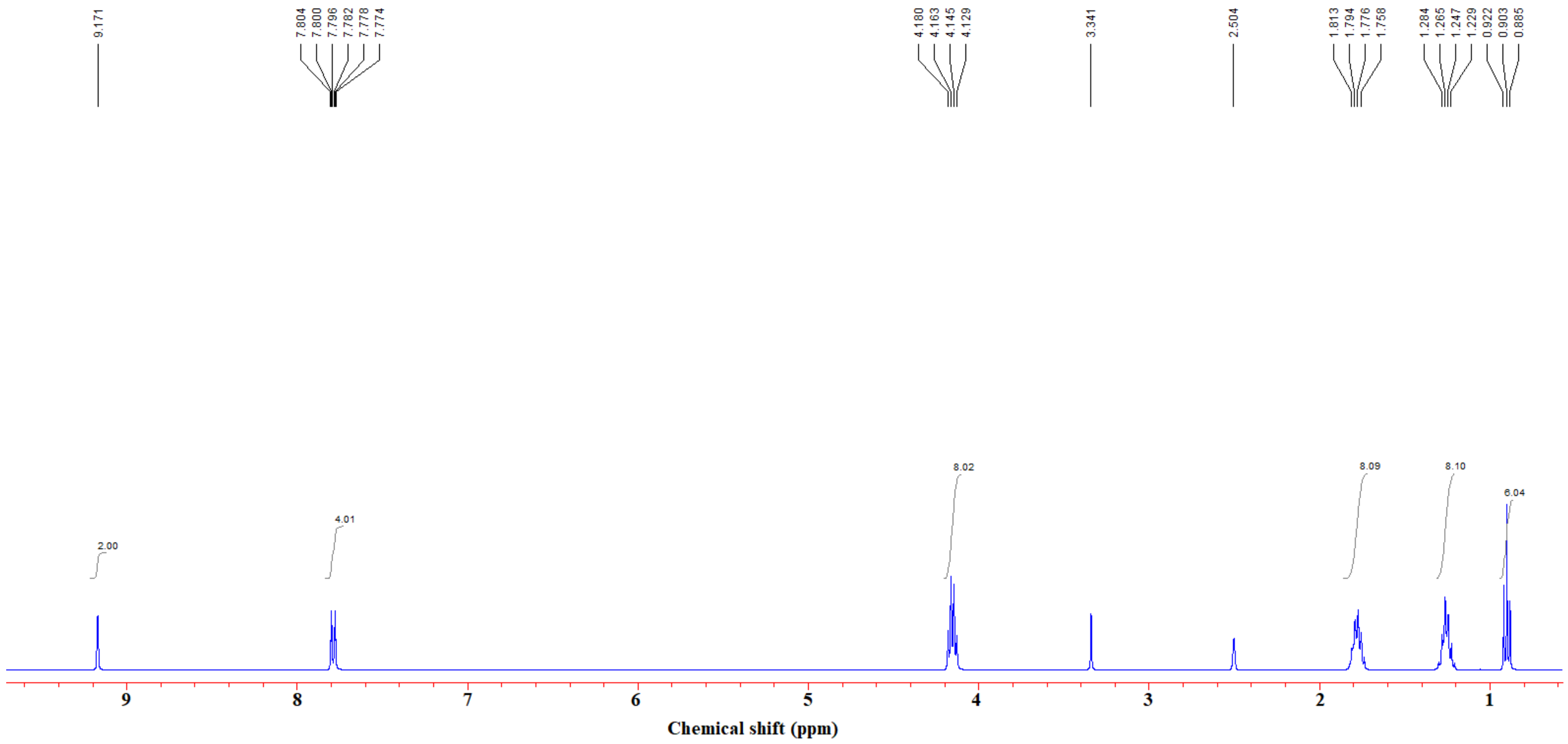

Figure S8. The ^{1}H NMR spectrum of $[C_6(Bim)_2](NTf_2)_2$ (400 MHz, DMSO-d_6).

Chemical shift (ppm): 0.885-0.922 (t, 6H), 1.229-1.284 (m, 8H), 1.758-1.813 (m, 8H), 4.129-4.180 (m, 8H), 7.774-7.804 (m, 4H), 9.171 (s, 2H).

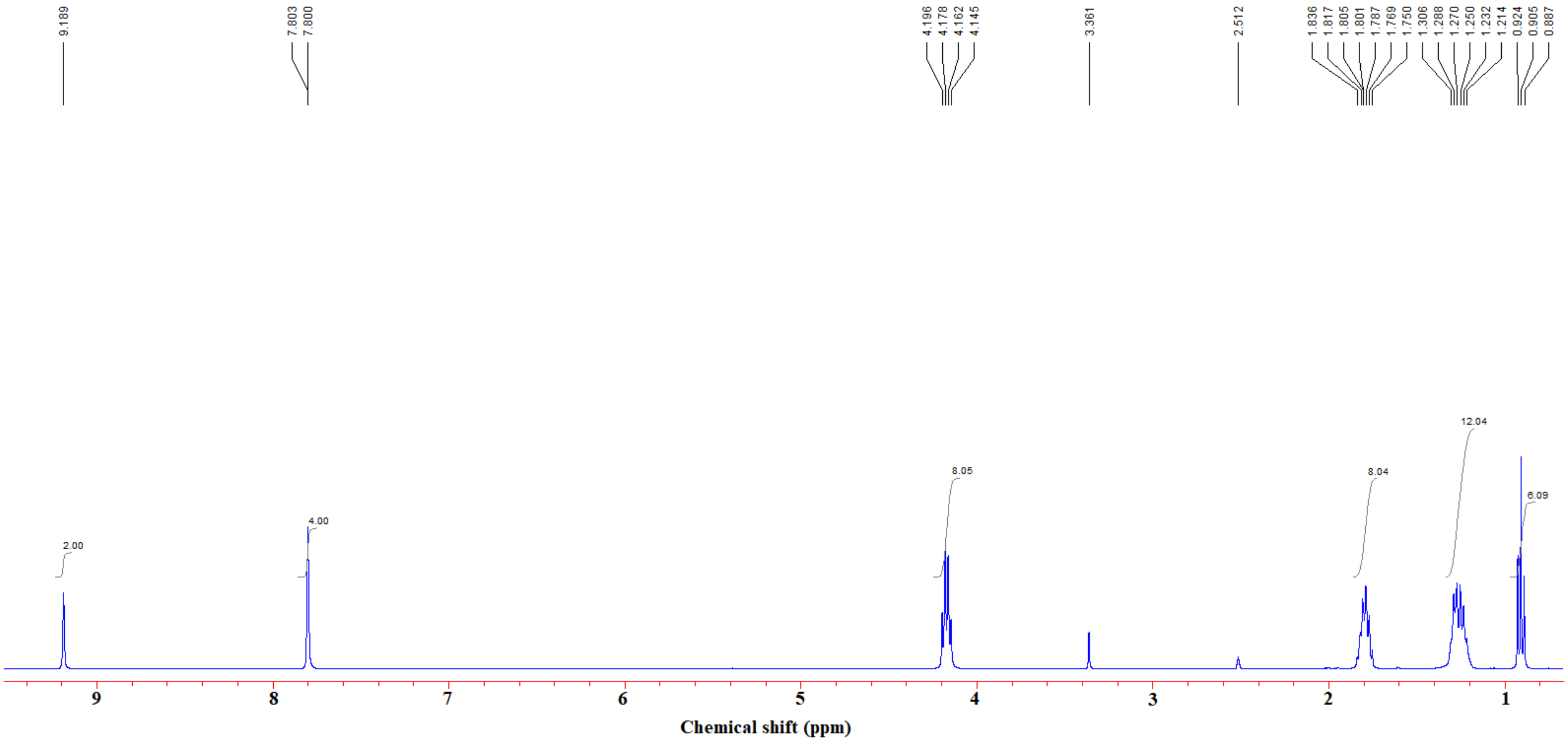

Figure S9. The ^{1}H NMR spectrum of $[C_8(Bim)_2](CF_3SO_3)_2$ (400 MHz, DMSO-d_6).

Chemical shift (ppm): 0.887-0.924 (t, 6H), 1.214-1.306 (m, 12H), 1.750-1.836 (m, 8H), 4.145-4.196 (m, 8H), 7.800-7.803 (d, 4H), 9.189 (s, 2H).

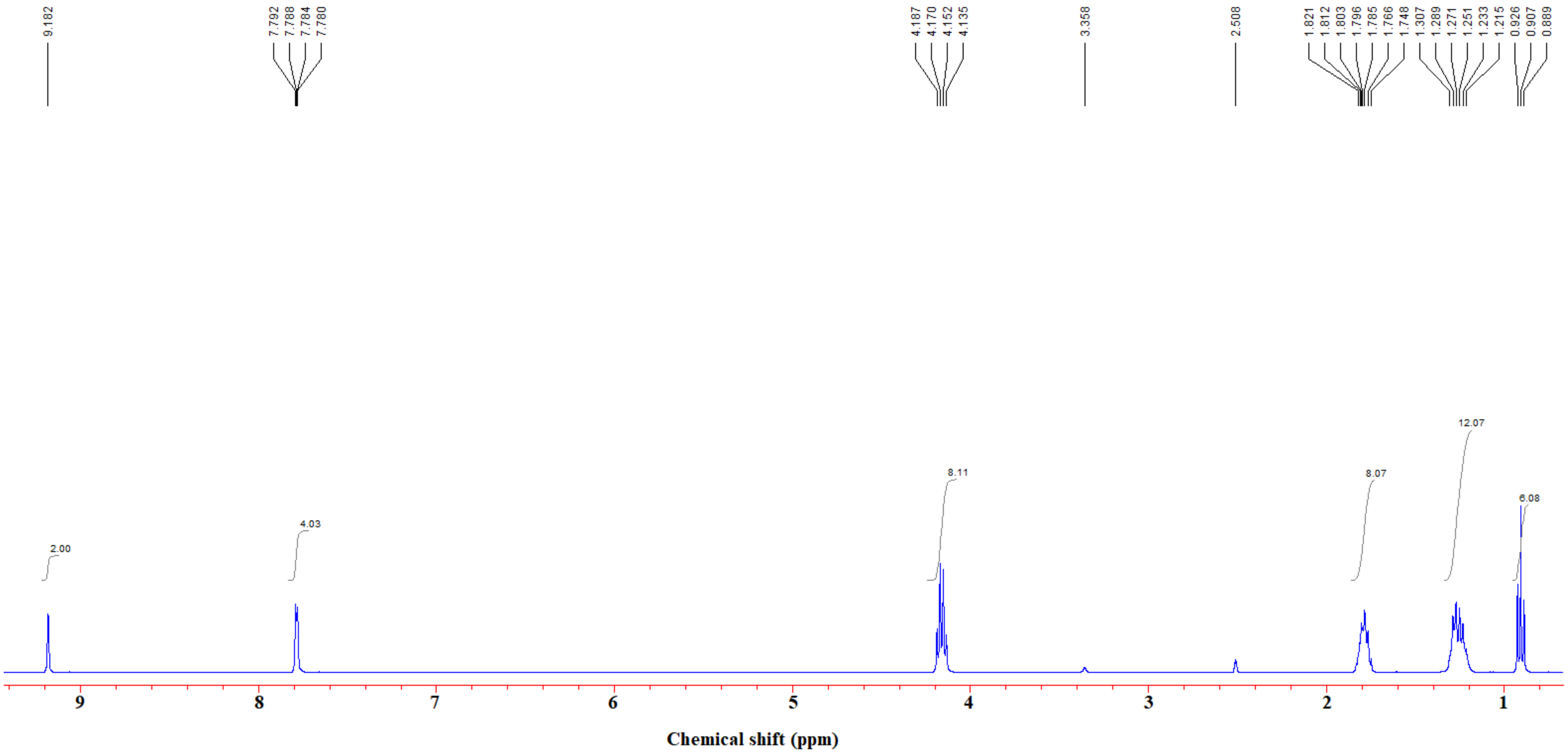

Figure S10. The ^{1}H NMR spectrum of $[C_8(Bim)_2](NTf_2)_2$ (400 MHz, DMSO-d_6).

Chemical shift (ppm): 0.889-0.926 (t, 6H), 1.215-1.307 (m, 12H), 1.748-1.821 (m, 8H), 4.135-4.187 (m, 8H), 7.780-7.792 (m, 4H), 9.182 (s, 2H).

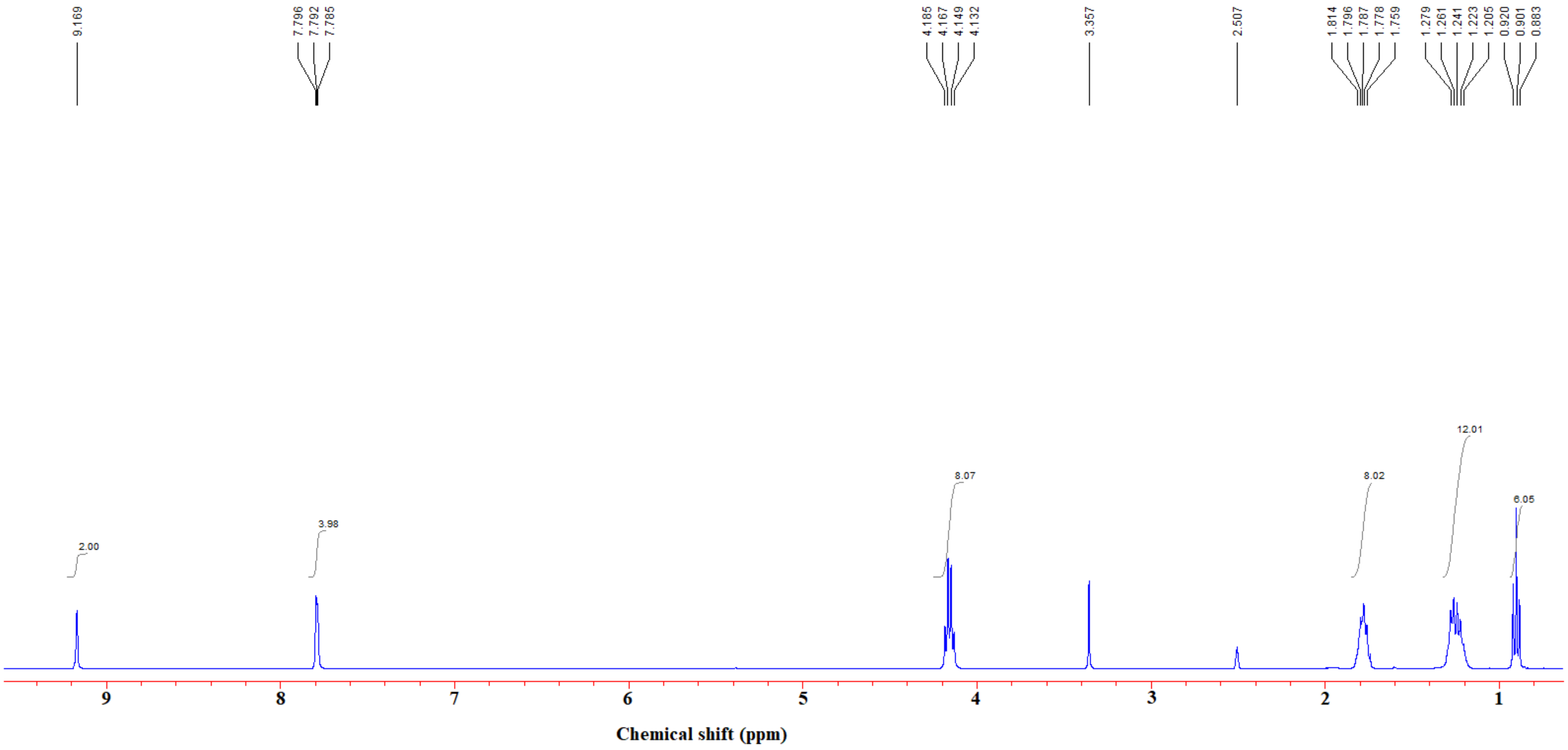

Figure S11. The ^{1}H NMR spectrum of $[C_8(Bim)_2](ClO_4)_2$ (400 MHz, DMSO-d_6).

Chemical shift (ppm): 0.883-0.920 (t, 6H), 1.205-1.279 (m, 12H), 1.759-1.814 (m, 8H), 4.132-4.185 (m, 8H), 7.785-7.796 (t, 4H), 9.169 (s, 2H).

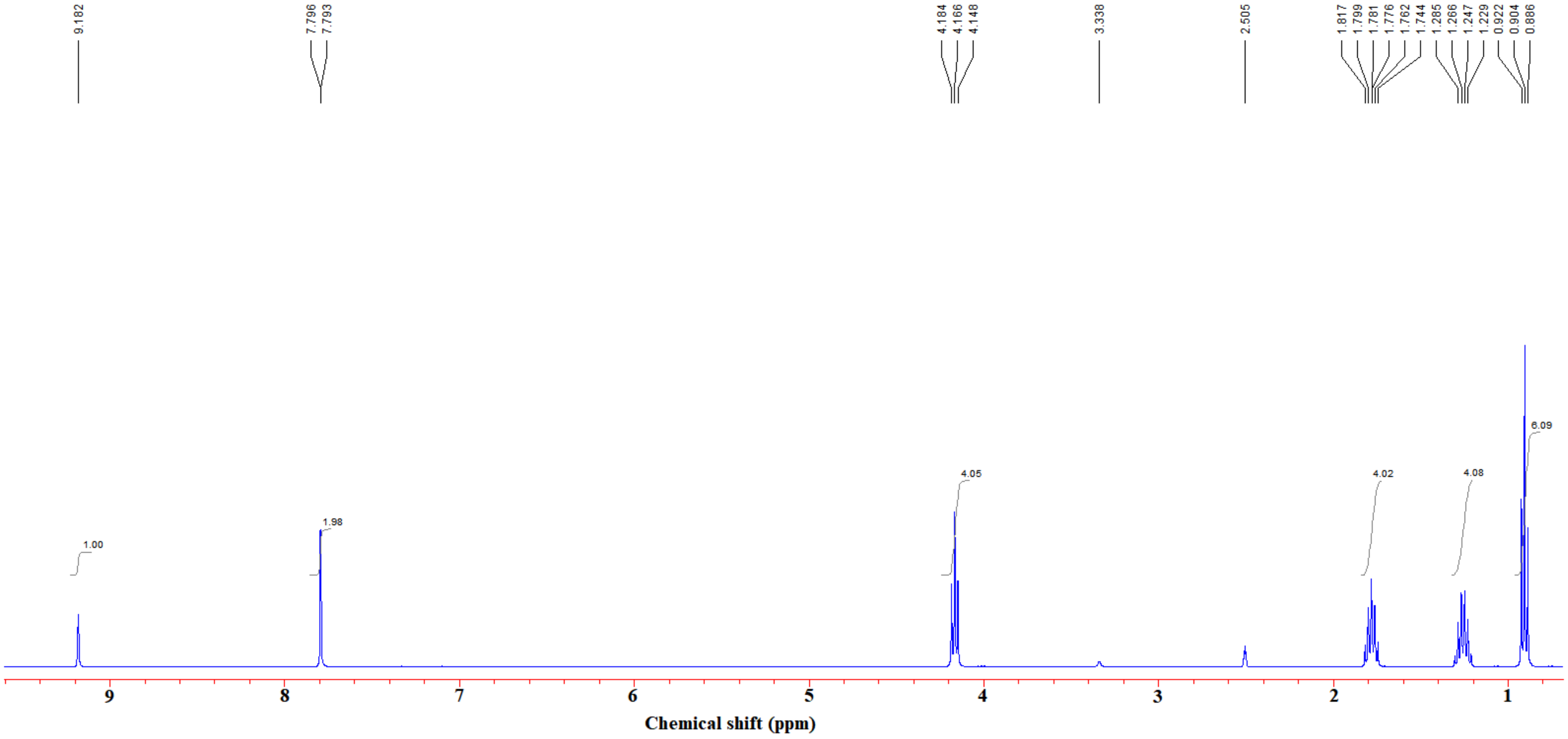

Figure S12. The ¹H NMR spectrum of [C_4Bim]ClO_4 (400 MHz, DMSO-d_6).

Chemical shift (ppm): 0.886-0.922 (t, 6H), 1.229-1.285 (m, 4H), 1.744-1.817 (m, 4H), 4.148-4.184 (t, 4H), 7.793-7.796 (d, 2H), 9.182 (s, 1H).

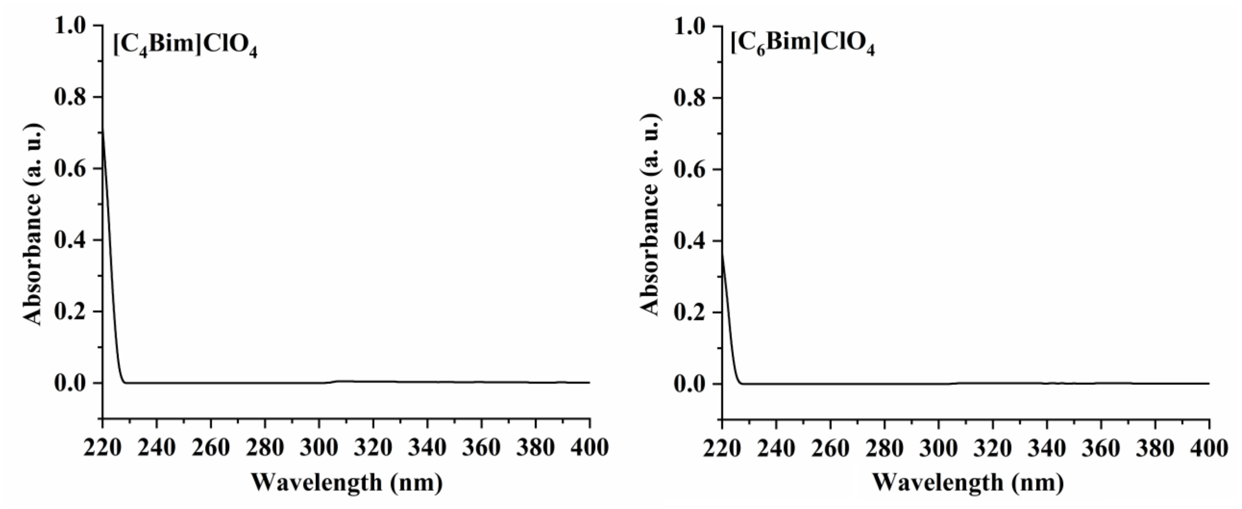

Figure S13. Ultraviolet absorption spectra of $[C_4Bim]ClO_4$ (0.23 g dm^{-3}) and $[C_6Bim]ClO_4$ (0.17 g dm^{-3}).

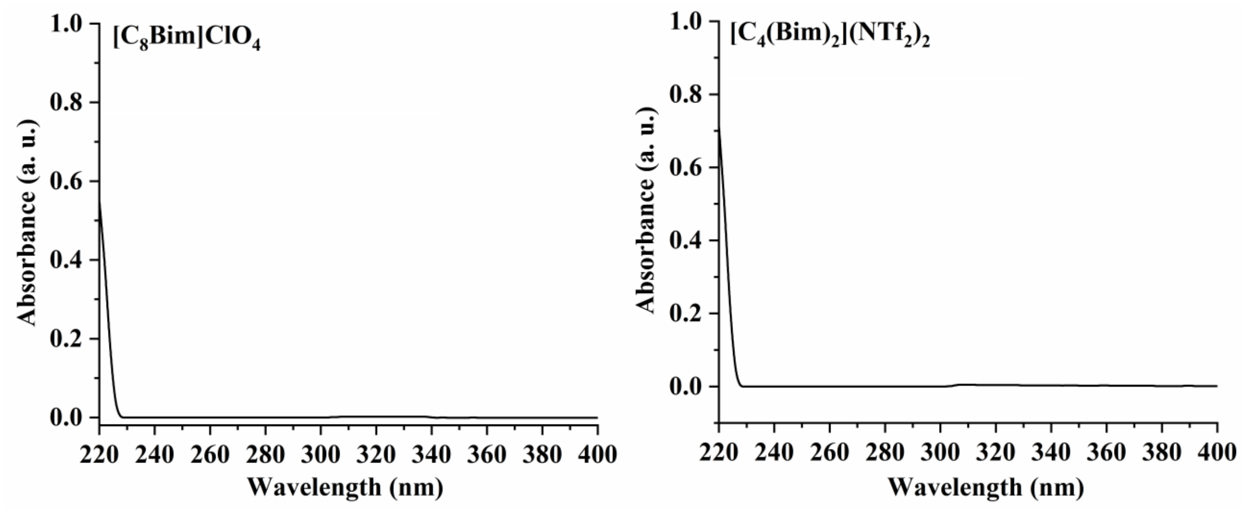

Figure S14. Ultraviolet absorption spectra of $[C_8Bim]ClO_4$ (0.13 g dm^{-3}) and $[C_4(Bim)_2](NTf_2)_2$ (0.32 g dm^{-3}).

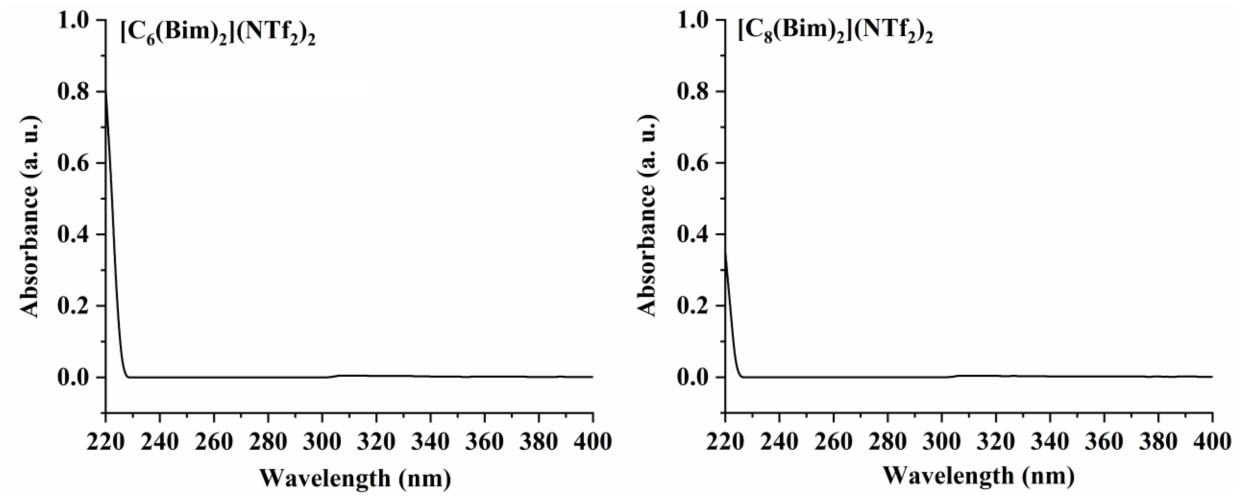

Figure S15. Ultraviolet absorption spectra of $[C_6(Bim)_2](NTf_2)_2$ (0.15 g dm^{-3}) and $[C_8(Bim)_2](NTf_2)_2$ (0.10 g dm^{-3}).

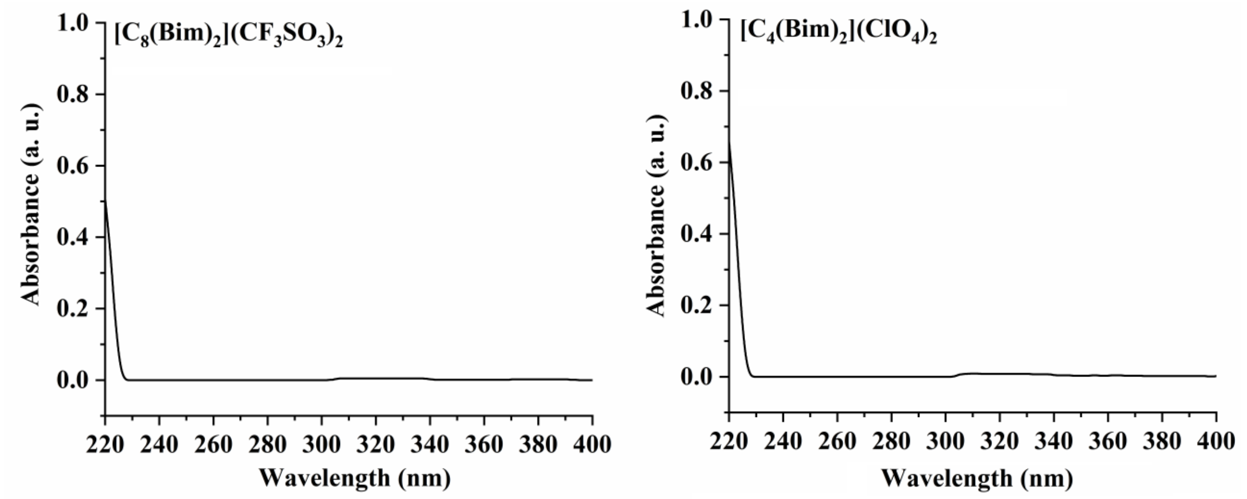

Figure S16. Ultraviolet absorption spectra of $[C_8(Bim)_2](CF_3SO_3)_2$ (0.14 g dm^{-3}) and $[C_4(Bim)_2](ClO_4)_2$ (0.11 g dm^{-3}).

Measurements of Hydrogen Bond Donating Ability and Hydrogen Bond Accepting Ability of the ILs

The hydrogen bond donating ability (α) and the hydrogen bond accepting ability (β) of the ILs used in this work were determined by using the three dyes, RD, DENA and NA as probes. The concentrations of DENA and NA in the ILs were both 3.0×10^{-5} mol dm^{-3} and the concentrations of RD in ILs were both 5.0×10^{-4} mol dm^{-3}. The maximum absorption wavelengths (λ_{max}) of the three dyes were determined by using a TU-1810 ultraviolet–visible spectrophotometer (Purkinje General Instrument Co., Beijing, China). The parameters, α and β are calculated using the following equations [1]:

$$\nu_{max}\ (cm^{-1}) = \frac{10^4}{\lambda(nm)} \quad (1)$$

$$E_T(30) = \frac{28592}{\lambda_{max}(nm, RD)} \quad (2)$$

$$\pi^* = 0.314\ (27.52 - \nu_{max}(DENA)) \quad (3)$$

$$\alpha = 0.0649\ E_T\ (30) - 2.03 - (0.72\pi^*) \quad (4)$$

$$\beta = \frac{1.035\ \nu_{max}(DENA) + 2.64 - \nu_{max}(NA)}{2.8} \quad (5)$$

Reference

1. Hauru, L.K.J.; Hummel, M.; King, A.; Kilpeläinen, I.; Sixta, H. Role of solvent parameters in the regeneration of cellulose from ionic liquid solutions. *Biomacromolecules* **2012**, *13,* 2896.
